# Pisa syndrome – a case report

**DOI:** 10.1192/j.eurpsy.2021.2116

**Published:** 2021-08-13

**Authors:** M. Bicho, J. Coelho, C. Peixoto, H. Fontes

**Affiliations:** Unidade De Agudos De Psiquiatria, Hospital do Divino Espírito Santo de Ponta Delgada, E.P.E., Ponta Delgada, Portugal

**Keywords:** Pisa Syndrome, Antipsychotics, dystonia, abnormal posture

## Abstract

**Introduction:**

Pisa Syndrome or pleurothotonus is a form of dystonia and often can arise as a side effect of antipsychotic treatment conditioning high morbidity and limiting management options. Despite the fact that the precise mechanism remains unclear, a neurochemical imbalance in dopaminergic and cholinergic transmission but also in serotoninergic and noradrenergic transmission can be a possible pathophysiologic mechanism, which can lead to changes in the axial axis with abnormal posture and marked lateral trunk flexion and abnormal gait.

**Objectives:**

Regarding a clinical case, the authors intend to review the relevant and current literature on the relationship between psychotropic drugs and Pisa Syndrome.

**Methods:**

Description of a clinical case by consulting databases of current and scientifically relevant articles.

**Results:**

The clinical case reports a 48-year-old woman with a history of HIV and Substance Use Disorder, hospitalized for unspecific behavioral changes, characterized by mood changes, self-referential, persecutory and somatic delusional ideas, and delusions of the control of thought. She was medicated with antipsychotics and mood stabilizers, with subsequent development of an acute-onset dystonic condition, characterizing the Pisa Syndrome. In this context, the dose of antipsychotics was lowered and anticholinergics were introduced, with progressive improvement of the clinical picture.

**Conclusions:**

Pisa Syndrome, previously seen as a rare adverse effect, can occur as a dystonic reaction related to the use of psychotropic drugs, so its use should be judicious. Further studies are needed to understand the extent of this association and its pathophysiological mechanisms in order to guide more rigorous therapeutic lines.

**Disclosure:**

No significant relationships.

